# New onset refractory status epilepticus (NORSE) versus refractory status epilepticus not meeting NORSE criteria: A comparative clinical and electroencephalography‐based study

**DOI:** 10.1002/epi.70384

**Published:** 2026-07-14

**Authors:** Seren Hawksworth, Nina Moutonnet, Gregory Scott, Sanjeev Rajakulendran, Mahinda Yogarajah, Matthew C. Walker, Umesh Vivekananda

**Affiliations:** ^1^ University College London Queen Square Institute of Neurology London UK; ^2^ Imperial College London UK Dementia Research Institute London UK; ^3^ Imperial College London London UK

**Keywords:** Electroencephalography, New‐onset refractory status epilepticus, Refractory status epilepticus, Status Epilepticus, Super‐refractory status epilepticus

## Abstract

**Objective:**

New onset refractory status epilepticus (NORSE) is a rare, severe presentation of refractory status epilepticus (RSE), with approximately half of cases cryptogenic NORSE (c‐NORSE). We compared electroencephalographic (EEG) findings alongside clinical features between NORSE and RSE not meeting NORSE criteria to better understand outcomes and prognostication.

**Methods:**

This retrospective cohort study analyzed 81 RSE patients admitted to the intensive therapy unit (ITU) at the National Hospital for Neurology and Neurosurgery and University College London Hospital (2007–2023). Patients were categorized as RSE not meeting NORSE criteria (*n* = 52) or NORSE (*n* = 29). EEG features, treatment, and outcomes (ITU stay, mortality, cognitive outcome, and antiseizure medication [ASM] use), were evaluated.

**Results:**

Of 29 NORSE patients (15 c‐NORSE, 51.7%; 14 etiology‐identified NORSE, 48.3%), autoimmune (20.7%) and infectious (17.2%) etiologies were most prevalent. Six‐month mortality was higher in c‐NORSE than etiology‐identified NORSE (20.0% vs. 7.1%), but similar between NORSE and RSE not meeting NORSE criteria (13.8% vs. 21.1%). EEG analysis revealed longer seizure duration (mean = 243.38 vs. 51.67 s, *p* < .001), a tendency to higher seizure frequency (.013 vs. .009 seizures/min, *p* = .076), and increased presence of generalized periodic epileptiform discharges and stimulus‐induced rhythmic, periodic, or ictal discharges in NORSE than RSE not meeting NORSE criteria. Across all patients, higher initial seizure burden correlated with longer ITU admissions and worse outcomes. Compared with RSE not meeting NORSE criteria, NORSE patients had longer ITU stays (median = 51 vs. 11 days, *p* < .001), more ASMs (mean = 3.91 vs. 2.00, *p* = .031), greater sedative use (mean = 2.17 vs. 1.00, *p* < .001), and more frequent cognitive impairment at discharge (88.9% vs. 62.2%, *p* = .037).

**Significance:**

NORSE, particularly c‐NORSE, tends to follow a more severe course than RSE not meeting NORSE criteria, with more severe background EEG features including the initial EEG, greater seizure burden, longer ITU stays, and increased incidence of cognitive impairment at discharge. Six‐month mortality was similar, implying additional contributing factors to mortality.

**Key points**

- NORSE, particularly cryptogenic NORSE, is more often associated with poor cognitive and functional outcomes compared with other causes of RSE.
- EEG features, such as initial seizure burden, seizure rate, and interictal patterns, may help differentiate between NORSE and RSE not meeting NORSE criteria.
- Early EEG monitoring (within 72 h of admission) may guide treatment escalation and improve prognosis.

## 1 INTRODUCTION

Refractory status epilepticus (RSE) occurs in approximately 30%–40% of status epilepticus (SE) patients and is associated with increased morbidity, and significantly higher mortality – nearly three times that of non‐refractory SE.^1^, ^2^ New onset refractory SE (NORSE) is a rare type of de novo RSE, in which there is no prior epilepsy history, and no cause identified within 48 h of presentation,^3^ which complicates management, because treatment is influenced by etiology.^4^ Furthermore, although an immune or infective etiology may be identified, nearly half of NORSE cases remain cryptogenic (cryptogenic NORSE [c‐NORSE]).^3^ NORSE management is challenging due to its heterogeneous causes and unclear mechanisms, resistance to antiseizure medications (ASMs), and limited evidence to guide treatment.^5^

Long‐term follow‐up data are limited; a mortality rate of 22% has been reported,^3^ with survivors of NORSE often facing cognitive impairment, physical disability, and drug‐resistant epilepsy.^6^, ^7^ Comparison of NORSE and RSE not meeting NORSE criteria subgroup outcomes has been contentious,^8^, ^9^ and critically, no specific electroencephalographic (EEG) markers differentiating NORSE from RSE not meeting NORSE criteria cases have been identified, hindering diagnostic precision and prognostic accuracy.

Although a number of studies have focused on the clinical features of NORSE to aid prognosis, management, and differentiation from RSE not meeting NORSE criteria,^6^, ^9^, ^10^ few have considered the impact of EEG findings. This study examines EEG findings and explores their roles in diagnosis and prognostication in NORSE.

## 2 MATERIALS AND METHODS

### 2.1 Study design

We conducted a retrospective observational cohort study to characterize and compare clinical, electrographic, and outcome variables in patients admitted with RSE to our hospitals. Patients were categorized into NORSE and RSE not meeting NORSE criteria groups. Within the NORSE group, cases were further divided into c‐NORSE and NORSE with an identified etiology (etiology‐NORSE) to enable subgroup analysis.

### 2.2 Patients, setting, and inclusion criteria

Patients admitted to the intensive therapy units (ITUs) of the National Hospital for Neurology and Neurosurgery or University College London Hospital (UCLH) between 2007 and 2023 were identified through a review of electronic health records (EHRs). Only cases of superrefractory SE (SRSE) were included, defined as SE persisting for >24 h after the initiation of anesthetic therapy or recurring upon its withdrawal. Patients were excluded if they were not in SE at the time of admission or if key clinical data (such as admission or discharge dates or ASM information) were unavailable. In total, 81 patients accounting for 95 intensive care admissions (14 of which were readmissions) with a diagnosis SRSE were included in the analysis.

Patients were grouped based on NORSE diagnostic criteria, defined as the absence of a prior epilepsy history and no identifiable structural, metabolic, or infectious cause within 48 h of admission (as per Gaspard et al.^3^). Patients who met these clinical criteria were diagnosed with NORSE in the ITU. NORSE patients were further subclassified as c‐NORSE if no cause could be identified throughout the admission, or etiology‐NORSE if a definitive diagnosis was established during the admission.

### 2.3 Ethical approval

Ethical approval was obtained from the UCLH Research Ethics Committee (Integrated Research Application System project ID: 305776, protocol number: 146147).

### 2.4 Clinical data collection and outcomes

Clinical data were extracted from the Trust's EHR system (EPIC), which provided inpatient medical notes, clinical EEG reports, discharge summaries, general practitioner referral letters, and neuropsychological assessments. All data were anonymized for analysis.

Clinical variables extracted included demographics, etiology (categorical, with 11 possible etiological groups), length of admission (continuous, days), epilepsy history (binary: yes/no), and the number of ASMs at discharge (count). Outcome variables included mortality at 6 months and 1 year postdischarge (binary: yes/no), cognitive impairment (binary: yes/no), and an overall outcome classification (binary: good/poor) assessed using data from the initial admission only, with readmissions excluded from outcome analyses. The overall outcome was classified as poor if the patient died or required full‐time care at any point within 1 year postdischarge, based on reports available within that period. Cognitive impairment was identified through clinical or neuropsychological assessments within 6 months postdischarge, marked as present if reports indicated decline from the patient's preadmission state.

### 2.5 EEG annotation data collection and analysis

EEG data were evaluated using clinical neurophysiology reports together with neurophysiologist annotations extracted from the Micromed clinical EEG database. The dataset included 2698 European Data Format (EDF) files totaling 12637.65 h of recording. The average EDF duration was 258.66 min (SD = 296.74). Annotations included time‐stamped markers of clinical and electrographic events observed during EEG recordings. Analysis focused on the presence of specific EEG features described in the clinical reports: generalized periodic discharges (GPDs); stimulus‐induced rhythmic, periodic, or ictal discharges (SIRPIDs); slow waves; and sharp waves (Table S1). EEG analyses were performed at two time points. First, the initial EEG, defined as the first EEG recorded within 72 h of admission or seizure onset, was analyzed separately to assess the prognostic value of early electrographic findings. Second, longitudinal EEG analyses included all available EEG recordings obtained throughout admission. The initial EEG was defined as the earliest EEG recorded for each patient. To ensure it reflected the early presentation, it was included only if performed within 72 h of hospital admission or, if no EEG was obtained on admission, within 72 h of the first documented seizure. The initial EEG was evaluated due to its potential prognostic value in guiding clinical management, its reflection of the early electrographic presentation, and its ability to provide insights into early treatment response.

Level of consciousness was recorded for each EEG from information documented in the neurophysiology reports and from details that could be interpreted, providing context of the patient's clinical state (Table S2). Seizure analysis involved reviewing the EEG annotations to determine seizure number and recording duration, allowing for calculation of seizure frequency per minute. From clinical EEG reports, the number and type of ASMs and sedatives administered during EEG monitoring were recorded. The average number of medications per patient per session was calculated, as well as the number of ASMs prescribed at discharge.

### 2.6 Statistical analysis

Statistical analyses were performed using IBM SPSS Statistics version 29. Comparisons between RSE not meeting NORSE criteria and NORSE, as well as between etiology‐NORSE and c‐NORSE, were made using the Mann–Whitney *U*‐test for continuous variables and Pearson chi‐squared test for categorical variables. Spearman rank correlation was used to assess associations between continuous seizure variables, clinical factors, and outcomes. Differences in drug administration were analyzed using chi‐squared tests. A *p*‐value of <.05 was considered statistically significant, with *p*‐values between .05 and .09 considered a trend. No correction for multiple comparisons was applied.

## 3 RESULTS

### 3.1 Patient characteristics

Patient characteristics for the dataset of 95 admissions, grouped into RSE not meeting NORSE criteria and NORSE, are summarized in Table 1. Among the SRSE cohort, 29 patients were classified as NORSE (16 female, mean age = 37.4 years), whereas 52 were classified as RSE not meeting NORSE criteria (31 female, mean age = 48.1 years). NORSE patients were significantly younger than RSE not meeting NORSE criteria patients (Mann–Whitney *U* = 515.5, *p* = .019). The readmission included 14 readmissions with RSE not meeting NORSE criteria. Time to first readmission ranged from 38 to 2410 days (median = 315, interquartile range [IQR] = 129–509). Of the 14 patients, eight were readmitted within 1 year. Across the readmissions, median length of admission was 23 days (SD = 37.65) and median length of ITU stay was 5.5 days (SD = 6.75).

**TABLE 1:** Characteristics of patients in RSE not meeting NORSE criteria and NORSE groups.

Characteristic	RSE not meeting NORSE criteria, *n* = 52	NORSE, *n* = 29	*p*
Female gender, *n* (%)	31 (59.6%)	16 (55.2%)	.698^a^
Age at admission, years, median	49.5 (minimum = 19, maximum = 85, IQR = 35)	31.0 (minimum = 18, maximum = 83, IQR = 24)	.**019** ^b^
Previous epilepsy, *n* (%)	24 (46.2%)	0 (0%)	**<.001** ^a^
Days of admission, median (IQR)^c^	25 (33)	94 (119)	**<.001** ^b^
Days of ITU stay, median (IQR)^c^	11 (15)	85 (102)	**<.001** ^b^

*Note*: Boldface indicates *p*‐value is considered significant.

Abbreviations: IQR, interquartile range; ITU, intensive therapy unit; NORSE, new onset refractory status epilepticus; RSE, refractory status epilepticus.

^a^ Chi‐squared test.

^b^ Mann–Whitney *U*‐test.

^c^ *n* = 95: NORSE, *n* = 29; RSE not meeting NORSE criteria, *n* = 66.

### 3.2 Etiology

Of the 29 NORSE cases, 15 (51.72%) were designated cryptogenic (c‐NORSE), where no definitive etiology could be determined despite extensive diagnostic evaluation (Table 2). Among the remaining 14 NORSE cases with etiologies eventually identified, autoimmune encephalitis (AIE) was most common. This included six cases of anti‐N‐methyl‐D‐aspartate receptor (NMDAR) encephalitis and one case of anti‐glutamic acid decarboxylase encephalitis. Other identified causes were herpes simplex virus encephalitis (four cases), Creutzfeldt–Jakob disease (one case, associated with a prion protein gene mutation), two paraneoplastic cases involving anti‐NMDAR antibodies linked to ovarian teratomas, and one brain neoplasm (not diagnosed within 48 h).

**TABLE 2:** Etiologies in NORSE and RSE not meeting NORSE criteria cases (*N* = 81).

Etiology	RSE not meeting NORSE criteria, *n* = 52, *n* (%)	NORSE, *n* = 29, *n* (%)
Cryptogenic	8 (15.4%)	15 (51.7%)
Autoimmune encephalitis	0 (0%)	6 (20.7%)
Infectious	4 (7.7%)	5 (17.2%)
Paraneoplastic	1 (1.9%)	2 (6.9%)
Brain neoplasm	5 (9.6%)	1 (3.4%)
Structural	13 (25.0%)	0 (0%)
Known epilepsy	10 (19.2%)	0 (0%)
Metabolic	6 (11.5%)	0 (0%)
Toxic	2 (3.8%)	0 (0%)
Alcohol withdrawal	1 (1.9%)	0 (0%)
Genetic	1 (1.9%)	0 (0%)
Inflammatory	1 (1.9%)	0 (0%)

*Note*: In RSE not meeting NORSE criteria, structural etiology here comprised *n* = 7 stroke, *n* = 4 traumatic brain injury, *n* = 1 hydrocephalus, and *n* = 1 edema from deep brain stimulation implantation. Autoimmune encephalitis included *n* = 5 anti‐N‐methyl‐D‐aspartate receptor encephalitis and *n* = 1 anti‐glutamic acid decarboxylase encephalitis. Paraneoplastic causes comprised *n* = 2 cases of encephalitis secondary to ovarian teratoma. Infectious etiologies included *n* = 4 herpes simplex encephalitis and *n* = 1 prion disease.

Abbreviations: NORSE, new onset refractory status epilepticus; RSE, refractory status epilepticus.

Among the 15 c‐NORSE patients, discharge documentation suggested that AIE was considered possible in five cases and a paraneoplastic syndrome in another, based on findings such as cerebrospinal fluid pleocytosis, positive response to immunotherapy, or the presence of an underlying tumor. However, due to the absence of confirmatory findings—such as seropositivity or definitive histopathological evidence—these cases remained undiagnosed and classified as cryptogenic for the purpose of the study. The remaining nine c‐NORSE cases had no suspected underlying cause.

In the 52 RSE not meeting NORSE criteria cases, structural causes were the most common, followed by established epilepsy without identified acute cause (Table 2). A subset of cases had no single clearly identified etiology; these patients often had multiple potential contributing factors, such as comorbidities or preexisting conditions, but no definitive cause could be determined. These cases were therefore not classified as NORSE, in contrast to c‐NORSE patients who presented without prior neurological conditions and for whom the etiology was entirely unknown.

### 3.3 Hospital admission

Across 95 admissions, patients with NORSE experienced significantly longer overall hospital stays compared to RSE not meeting NORSE criteria patients. The median length of admission was 94 days (IQR = 102) for NORSE versus 27 days (IQR = 34) for RSE not meeting NORSE criteria (*U* = 308.50, *p* < .001). ITU stays were also significantly prolonged in NORSE cases, with a median of 85 days (IQR = 102) compared to 11 days (IQR 15) in RSE not meeting NORSE criteria (*U* = 144.50, *p* < .001). NORSE ITU stays ranged widely from 9 to 522 days, whereas RSE not meeting NORSE criteria ranged from 1 to 81 days.

### 3.4 Treatment

Data on ASM and sedative use were available for 61 admissions. For each admission, the number of ASMs and sedatives (intravenous) documented during EEG sessions was averaged, and the median of these averages was compared between groups. Median ASM usage was higher in NORSE compared to RSE not meeting NORSE criteria (*U* = 264.00, *p* = .050; Table 3), with sedative use also significantly greater in the NORSE group (*U* = 147.00, *p* < .001). c‐NORSE patients required more ASMs during the admission than etiology‐NORSE (*U* = 18.50, *p* = .070; Table 3). For non‐ASM treatment, plasma exchange and steroids were administered to 48.3% of NORSE patients, whereas intravenous immunoglobulin and antivirals were administered to 37.9%. All rituximab treatments in etiology‐NORSE were for AIE. Additional therapies included anakinra, cannabidiol, and ketogenic diet, which was implemented in 6.9% of NORSE cases, exclusively within the c‐NORSE subgroup. Among the 28 NORSE cases with available data, 79.3% had an infectious prodrome, and 89.7% were previously healthy. A total of 73.3% of c‐NORSE had an infectious prodrome, compared to 85.7% of etiology NORSE (83.3% of AIE, 100% of infectious, 100% of paraneoplastic, 0% of brain neoplasm).

**TABLE 3:** Average number of ASMs and sedatives throughout the admission in RSE not meeting NORSE criteria and NORSE cases.

	RSE not meeting NORSE criteria, *n* = 43	NORSE, *n* = 18	*p*
Average number of ASMs	2.00 (3.00), maximum = 7.14	3.86 (1.89), maximum = 7.00	.**050** ^a^
Average number of sedatives	1.00 (1.00), maximum = 2.75	2.23 (1.07), maximum 3.56	**<.001** ^a^

*Note*: Values are median (interquartile range). Boldface indicates *p*‐value is considered significant. Abbreviations: ASM, antiseizure medication; NORSE, new onset refractory status epilepticus; RSE, refractory status epilepticus.

^a^ Mann–Whitney *U*‐test.

ASM administration was recorded for each EEG session; across all EEGs, levetiracetam was given in 91.7%, phenytoin in 48.3%, and sodium valproate in 33.3%. Several ASMs were significantly more commonly used in NORSE patients compared to RSE not meeting NORSE criteria, including sodium valproate (χ^2^[1] = 8.93, *p* = .003), phenytoin (χ^2^[1] = 8.93, *p* = .003), perampanel (χ^2^[1] = 11.71, *p* < .001), diazepam (χ^2^[1] = 11.71, *p* < .001), lacosamide (χ^2^[1] = 5.73, *p* = .017), vigabatrin (χ^2^[1] = 4.83, *p* = .028), and phenobarbital (χ^2^[1] = 8.90, *p* = .003). Furthermore, longer ITU stays correlated with a greater number of ASMs per EEG throughout admission (rho = .407, *p* = .001) and a greater number of sedatives (rho = .493, *p* < .001).

Regarding sedatives, fentanyl (55.0%), propofol (51.7%), and midazolam (30.0%) were the most frequently administered agents. Propofol, ketamine, thiopental, fentanyl, midazolam, and lorazepam were significantly more common in the NORSE group than in RSE not meeting NORSE criteria (χ^2^[1] = 14.37, *p* < .001; χ^2^[1] = 7.37, *p* = .007; χ^2^[1] = 4.83, *p* = .028; χ^2^[1] = 11.93, *p* < .001; χ^2^[1] = 28.00, *p* < .001, respectively), whereas lorazepam was administered more frequently in the RSE not meeting NORSE criteria group (χ^2^[1] = 5.77, *p* = .016).

### 3.5 EEG analysis

Among recordings with a reported location, 89.92% were performed in an ITU, predominantly within the Medical ITU (67.9%), a specialized neurological ITU, followed by ward‐based EEGs (6.7%) and inpatient neurophysiology settings (3.38%). The majority of recordings (97.62%) were reported as abnormal in clinical reports, with abnormalities more frequently identified in NORSE (99.44%) than RSE not meeting NORSE criteria (94.12%).

### 3.6 First EEG

The first clinical EEG, defined as the first recording performed within 72 h of admission or the initial seizure, was analyzed, excluding 24 individuals who did not have an EEG available within this timeframe; there was no significant difference in first EEG duration between NORSE and RSE not meeting NORSE criteria patients (mean duration = 90.52 min, SD = 229.48, *U* = 294.0, *p* = .113). Consequently, the analyzed cohort comprised patients with RSE not meeting NORSE criteria (*n* = 54) and NORSE (*n* = 17). Seizure activity, as documented in the EEG reports, was present in 29.0% of initial EEGs. This was significantly more frequent in NORSE compared to RSE not meeting NORSE criteria (52.94% vs. 21.28%, χ^2^ = 6.289, *p* = .012). The median duration of seizures on the initial EEG was 125.67 s (IQR = 233.23), with longer durations observed in NORSE patients compared to RSE not meeting NORSE criteria (239.25 s vs. 74.0 s, *p* = .083, *U* = .000).

NORSE patients exhibited more seizures per minute (rate) on the first EEG compared to RSE not meeting NORSE criteria (median [SD] = .013 [.029] vs. .009 [.039], *U* = 326.00, *p* = .076). Across all RSE admissions, higher seizure rates in the initial EEG correlated with longer ITU stays (*n* = 62, rho = .270, *p* = .034) and more ASMs throughout admission (*n* = 46, rho = .320, *p* = .030). Neurophysiology reports showed NORSE cases had higher incidences of interictal epileptiform discharges (100% vs. 54.1%, χ^2^[1] = 11.258, *p* < .001), sharp waves (87.5% vs. 54.9%, χ^2^[1] = 7.22, *p* = .007), and GPDs (12.5% vs. .0%, χ^2^[1] = 5.56, *p* = .018) than RSE not meeting NORSE criteria. NORSE cases also received phenobarbital (χ^2^[1] = 91.09, *p* < .001), midazolam (χ^2^[1] = 94.80, *p* < .001), and propofol (χ^2^[1] = 78.83, *p* < .001) significantly more often in the initial EEG than RSE not meeting NORSE criteria.

### 3.7 EEG analysis across admission

Seizure rate was calculated by dividing the total number of seizures by the total duration of the EEG recording (in minutes). The median seizure rate was .09 seizures per minute (IQR = .22) in RSE not meeting NORSE criteria and .32 seizures per minute (IQR = .27) in NORSE, with NORSE patients having significantly more seizures per EEG throughout admission than RSE not meeting NORSE criteria (χ^2^[1] = 18.47, *p* < .001; Table 4).

**TABLE 4:** Comparison of EEG patterns across admission between RSE not meeting NORSE criteria and NORSE.

	RSE not meeting NORSE criteria	NORSE	*p*
Rhythmic or periodic patterns, *n* (%)	*n* = 52	*n* = 18	
PLDs	2 (3.8%)	3 (16.7%)	.069^a^
GPDs	1 (1.9%)	9 (50%)	**<.001** ^a^
SIRPIDs	0 (0%)	2 (11.1%)	.**015** ^a^
EEG features, *n* (%)	*n* = 66	*n* = 18	
Burst suppression	5 (7.7%)	12 (66.7%)	**<.001** ^a^
Severe encephalopathy^b^	5 (7.57%)	9 (47.4%)	**<.001** ^a^
Slowing	59 (89.4%)	18 (100.0%)	.149^a^
Seizures, median (IQR)	*n* = 7	*n* = 8	
Seizures per minute^c^	.09 (.22)	.32 (.27)	**<.001** ^d^
Maximum seizure	77.00 (664.00)	532.00 (7656.50)	.125^d^
Mean seizure	63.33 (285.37)	99.04 (274.32)	.266^d^

*Note*: Boldface indicates *p*‐value is considered significant.

Abbreviations: EEG, electroencephalographic; GPD, generalized periodic discharge; IQR, interquartile range; NORSE, new onset refractory status epilepticus; RSE, refractory status epilepticus; SIRPID, stimulus‐induced rhythmic, periodic, or ictal discharge.

^a^ Chi‐squared test.

^b^ *n* = 85 (RSE not meeting NORSE criteria, *n* = 66; NORSE, *n* = 19).

^c^ *n* = 84 (RSE not meeting NORSE criteria, *n* = 62; NORSE, *n* = 22). Reported as mean (SD).

^d^ Mann–Whitney *U*‐test.

EEG patterns such as SIRPIDs, GPDs, and burst suppression were significantly more common in NORSE than RSE not meeting NORSE criteria (χ^2^[1] = 5.95, *p* = .015; χ^2^[1] = 25.38, *p* < .001; χ^2^[1] = 30.59, *p* < .001), with periodic lateralised discharges (PLD) presence correlating with poor outcome (χ^2^[1] = 3.26, *p* = .071).

Higher seizure rates across admission correlated with longer admission (rho = .425, *p* < .001), as well as more ASMs (rho = .577, *p* < .001) and sedatives (rho = .378, *p* = .004) administered. Patients with higher seizure rates were also discharged on a greater number of ASMs (rho = .455, *p* < .001) and were more likely to exhibit cognitive impairment postdischarge (*U* = 203.50, *p* = .003).

Across SRSE, higher maximum seizure length correlated with longer ITU stay (rho = .483, *p* = .058) and more sedatives (rho = .708, *p* = .002). Longer seizure length was correlated with longer ITU stay (rho = .480, *p* = .060) and higher average sedative use (rho = .649, *p* = .007). NORSE patients had significantly higher seizure rate than RSE not meeting NORSE criteria patients (*U* = 325.00, *p* < .001), but no notable difference in maximum or mean seizure length.

### 3.8 Patient outcomes

Among 81 patients (excluding readmission cases), 15 (18.5%) died either during admission or within 6 months postdischarge (Table 5). Of these, 10 deaths occurred in the ITU: eight patients with RSE not meeting NORSE criteria (including three with structural causes, two with brain neoplasms, one paraneoplastic, one with known epilepsy, and one with alcohol withdrawal) and two patients with true NORSE.

**TABLE 5:** Clinical outcomes in RSE not meeting NORSE criteria and NORSE cases in base patients.

	RSE not meeting NORSE criteria, *n* = 52	NORSE, *n* = 29	*p*
Poor outcome^a^	24 (46.2%)	11 (39.3%)	.555^b^
Deceased within 6 months	11 (21.1%)	4 (13.8%)	.521^b^
Cognitive impairment^c^	28 (62.2%)	16 (88.9%)	.**037** ^b^
ASMs on discharge^d^	.73 (1.40)	3.07 (1.96)	**<.011** ^e^

*Note*: Values are *n* (%), except ASMs on discharge, reported as mean (SD). Boldface indicates *p*‐value is considered significant.

Abbreviations: ASM, antiseizure medication; ITU, intensive therapy unit; NORSE, new onset refractory status epilepticus; RSE, refractory status epilepticus.

^a^ *n* = 81 (RSE not meeting NORSE criteria, *n* = 52; NORSE, *n* = 29) due to lack of follow‐up information.

^b^ Chi‐squared test.

^c^ *n* = 63 (RSE not meeting NORSE criteria, *n* = 45; NORSE, *n* = 18) due to a lack of reports and patients who died in the ITU.

^d^ *n* = 71 (RSE not meeting NORSE criteria, *n* = 44; NORSE, *n* = 27) due to 10 patients dying in the ITU.

^e^ Mann–Whitney *U*‐test.

The etiologies among those who died either during admission or within 6 months postdischarge included four with NORSE (three cryptogenic and one due to prion disease) and 11 with RSE not meeting NORSE criteria (five structural, three brain neoplasms, one paraneoplastic, one with known epilepsy, and one with alcohol withdrawal).

Overall, 35 patients (43.2%) experienced poor outcomes, and 44 (69.8%) were documented as having postdischarge cognitive impairment based on discharge summaries and formal cognitive assessments. Cognitive impairment was significantly more prevalent in NORSE than RSE not meeting NORSE criteria (88.9% vs. 62.2%; χ^2^[1] = .037, *p* = .037), consisting mainly of widespread cognitive dysfunction, memory problems, and executive dysfunction. Higher age at admission was associated with poorer outcome (*U* = 478.50, *p* = .006) and increased 6‐month mortality (*U* = 241.50, *p* = .003). When analyzing etiology, brain neoplasms and structural causes were linked to higher 6‐month mortality when compared to all other etiologies (χ^2^[1] = 4.06, *p* = .045; χ^2^[1] = 4.73, *p* = .030).

Across all patients, longer ITU stay was associated with higher 6‐month mortality (*U* = 318.00, *p* = .043), particularly in NORSE (*U* = 12.00, *p* = .013). Prior epilepsy was associated with shorter hospital (*U* = 375.50, *p* = .004) and ITU stays (*U* = 384.50, *p* = .006), as well as lower sedative use (*U* = 207.00, *p* = .077), but did not correlate with outcome. Patients with better outcome received more ASMs during the first EEG (*U* = 333.50, *p* = .052), particularly in NORSE patients (*U* = 8.50, *p* = .003). In NORSE, higher average number of sedatives use across admission was also correlated with better outcome (*U* = 12.00, *p* = .025). At discharge, NORSE patients were prescribed significantly more ASMs than RSE not meeting NORSE criteria (*U* = 170.50, *p* = .011). Among the 27 surviving NORSE patients, 92.6% required ASMs on discharge, significantly higher than RSE not meeting NORSE criteria (34.1%, χ^2^[1] = 26.86, *p* < .001).

### 3.9 NORSE subcategorization

Distinct clinical outcomes and treatment patterns were observed between c‐NORSE and etiology‐NORSE, with c‐NORSE associated with poorer outcome (χ^2^[1] = 3.74, *p* = .053) (Table 6). Six‐month mortality was 12.9% higher in c‐NORSE than in etiology‐NORSE. Additionally, c‐NORSE patients required significantly more ASMs at discharge than etiology‐NORSE (*U* = 43.00, *p* = .006). The presence of PLD was also linked to poor outcomes in NORSE (χ^2^[1] = 4.67, *p* = .031).

**TABLE 6:** Comparison of clinical features, treatment, outcome, and hospital stay between c‐NORSE and etiology NORSE.

	NORSE	c‐NORSE	Etiology‐NORSE	*p*
Clinical features, *n* (%)	*n* = 29	*n* = 15	*n* = 14	
Infectious prodrome	23 (79.3%)	11 (73.3%)	12 (85.7%)	.245^a^
Treatment, *n* (%)	*n* = 29	*n* = 15	*n* = 14	
Ketogenic diet	2 (6.9%)	2 (13.3%)	0 (.0%)	.214^a^
Rituximab	5 (17.2%)	2 (13.3%)	3 (21.4%)	.538^a^
IVIG	12 (41.4%)	7 (46.7%)	4 (28.6%)	.398^a^
Antivirals	12 (41.4%)	6 (40.0%)	6 (42.8%)	.354^a^
Plasma exchange	14 (48.3%)	7 (46.7%)	7 (50.0%)	.563^a^
Steroids	14 (48.3%)	8 (53.3%)	6 (42.9%)	.573^a^
Outcome, *n* (%)	*n* = 29	*n* = 15	*n* = 14	
Cognitive impairment^b^	15 (88.2%)	5 (83.3%)	10 (90.9%)	.732^a^
Deceased in 6 months	4 (13.8%)	3 (20.0%)	1 (7.1%)	.316^a^
Deceased in 1 year	5 (17.2%)	4 (26.7.%)	1 (7.1%)	.164^a^
Poor outcome^c^	11 (39.3%)	8 (57.1%)	3 (21.4%)	.053^a^
ASMs on discharge^d^	3.07 (1.96)	4.31 (1.49)	1.93 (1.64)	.**006** ^e^
Hospital stay, median (IQR)	*n* = 29	*n* = 15	*n* = 14	
Days of admission	80.5 (148)	94.0 (143)	63.0 (163)	.310^e^
Days of ITU stay	73.5 (138)	94.0 (134)	51.0 (173)	.533^e^
ASMs across admission^f^	3.86 (2.12)	4.10 (2.38)	3.00 (2.91)	.069^e^
Sedatives across admission^f^	2.23 (1.47)	2.44 (.67)	.88 (2.86)	.211^e^

*Note*: Boldface indicates *p*‐value is considered significant.

Abbreviations: ASM, antiseizure medication; c‐NORSE, cryptogenic NORSE; IQR, interquartile range; ITU, intensive therapy unit; IVIG, intravenous immunoglobulin; NORSE, new onset refractory status epilepticus.

^a^ Chi‐squared test.

^b^ *n* = 17 (c‐NORSE, *n* = 6; etiology‐NORSE, *n* = 11) due to the lack of availability of neuropsychology reports and patients dying in hospital.

^c^ *n* = 28 (c‐NORSE, *n* = 14; etiology‐NORSE, *n* = 14) due to lack of follow‐up information.

^d^ *n* = 27 (c‐NORSE, *n* = 13; etiology‐NORSE, *n* = 14) due to two c‐NORSE patients dying in hospital. Reported as mean (SD).

^e^ Mann–Whitney *U*‐test.

^f^ *n* = 18 (c‐NORSE, *n* = 11; etiology‐NORSE, *n* = 7). Reported as mean (SD).

## 4 DISCUSSION

This study aimed to characterize our SRSE cohort, and to compare clinical and EEG features of NORSE with RSE not meeting NORSE criteria. Outcome in NORSE was associated with the eventual identification of an etiology, with c‐NORSE demonstrating poorer outcomes than those with an identified underlying cause. Treatment challenges were more pronounced in cryptogenic cases, where the lack of a defined etiology may have limited the ability to implement targeted interventions and achieve seizure control, as reflected in prolonged ITU admissions, higher seizure burden, and increased reliance on ASMs and sedatives. EEG findings further distinguished NORSE from RSE not meeting NORSE criteria, with higher initial seizure rates, longer median seizure durations, and more frequent GPDs and sharp waves. These initial EEG features provide early indicators of disease severity and prognosis, with higher seizure rates on the first EEG correlating with longer ITU stays and increased ASM use across admission.

### 4.1 Etiology and prognosis

A total of 51.7% of NORSE cases remained cryptogenic, consistent with previous studies.^3^ AIE was eventually identified in 19% of NORSE cases, in line with emerging evidence of immune‐mediated contributions in NORSE.^10^, ^11^ NORSE mortality 6 months postdischarge was 13.8%, lower than the 22% reported previously.^3^ Poor outcomes occurred in 39.3% of NORSE cases, with significantly higher rates in c‐NORSE than etiology‐NORSE, emphasizing the value of comprehensive diagnostic evaluation. Reduced reliance on ASMs in etiology‐NORSE further highlights how early identification can guide targeted treatments, such as immunotherapy. Higher seizure rates on the first EEG and across admission correlated with longer ITU stays, higher sedative use, greater ASM requirements, and higher prevalence of cognitive impairment at discharge.

### 4.2 Clinical characteristics

A history of epilepsy (observed in RSE not meeting NORSE criteria) was associated with shorter ITU stays and lower ASM and sedative requirements, suggesting reduced refractoriness and faster clinical recovery. To note, in this study these patients were not classified as febrile infection‐related epilepsy syndrome (FIRES), a subtype of NORSE in which SRSE can develop following a prior febrile illness.^12^ Patients were clinically diagnosed as NORSE, and because the cohort spans 2007–2023—prior to widely accepted consensus criteria for FIRES—subtyping within NORSE was not possible.

### 4.3 Treatment and management

NORSE patients experienced extended ITU stays, as well as longer sedative use and more ASMs during admission and at discharge than RSE not meeting NORSE criteria cases, likely reflecting prolonged duration and severity of SE. Despite immunotherapy and progressively escalating ASM regimens, NORSE, particularly c‐NORSE, continued to demonstrate poor seizure control, requiring multiple treatments without achieving cessation of SE. EEG patterns found that higher seizure rates across admission correlated with greater ASM and sedative use.

### 4.4 Long‐term outcomes

Long‐term outcomes of NORSE include the development of epilepsy and cognitive impairment.^7^ In our cohort, 39.3% of NORSE patients experienced poor outcomes, defined as death or severe disability, with a higher prevalence in c‐NORSE compared to etiology‐identified NORSE. Cognitive impairment was observed in 88.9% of NORSE patients, versus 62.2% in RSE not meeting NORSE criteria, likely reflecting the cumulative impact of prolonged ITU admission, pharmacological interventions, and underlying pathology. Higher seizure rates and longer seizure durations recorded on EEGs across admission correlated with increased ASM prescription at discharge and cognitive impairment, highlighting EEG as a valuable prognostic tool.

### 4.5 EEG characteristics and prognostic value

NORSE patients exhibited longer seizure durations, higher maximum seizure duration, and more seizures per minute than RSE not meeting NORSE criteria. The occurrence of GPDs and SIRPIDs was higher in the NORSE subgroup. These EEG findings may reflect a stronger seizure liability in NORSE, or alternatively, be indicative of a more pronounced encephalopathic state. The presence of PLDs was associated with poorer outcomes, supporting their use as markers of ongoing cerebral dysfunction and seizure burden.^13^, ^14^

From the initial EEG, a higher seizure rate was significantly associated with longer hospital and ITU stays, as well as poorer clinical outcomes. Longer initial seizure duration similarly correlated with extended ITU admission and greater sedative use, highlighting that initial seizure burden may indicate disease severity and treatment refractoriness. Conversely, administration of more ASMs during the initial EEG was linked to better outcomes in both RSE overall and the NORSE subgroup. However, as this is a retrospective observational study, these associations may reflect treatment indication rather than a direct therapeutic effect. Furthermore, preadmission ASM use was not systematically collected and may represent an additional confounding factor, as patients with established epilepsy receiving ASM therapy prior to RSE onset may differ in their electroclinical presentation and outcomes compared with those not receiving treatment at seizure onset. Likewise to ASMs, increased initial sedative use in NORSE patients demonstrated a comparable association with better outcomes, implying that early escalation may improve prognosis. These findings suggest that early EEG assessment can provide prognostic information.

### 4.6 Limitations

The retrospective design of the study posed several challenges, including limited follow‐up data, absence of pre‐ITU admission details, and incomplete or missing medical records. The study relied heavily on the availability, accuracy, and organization of these records. The relatively small dataset limits the generalizability of these findings and precludes definitive conclusions, emphasizing the need for larger multicenter studies to validate and expand upon these observations. Categorization of EEGs was challenging, as neurophysiology reports were often overly succinct, summarizing complex continuous EEG data into a single paragraph. Variability in EEG reports introduced an increased risk of missed or misinterpreted electrophysiological events, and many seizures were likely unreported.

## 5 CONCLUSIONS

This study provides insights into clinical and EEG features of RSE and underscores the challenges of NORSE. Compared to RSE not meeting NORSE criteria, NORSE was more frequently associated with poor cognitive and functional outcomes, emphasizing the need for early etiology identification and improved therapeutic strategies. The prognostic value of EEG was demonstrated by associations of EEG‐derived initial seizure burden, seizure rate, and abnormalities such as PLDs with the clinical features of longer hospital stays, higher sedative requirements, and poorer outcomes, highlighting the potential benefit of early EEG monitoring for guiding treatment escalation and prognosis.

## AUTHOR CONTRIBUTIONS

Seren Hawksworth contributed to conceptualization and study design, data collection, and data analysis. Gregory Scott contributed to conceptualization, EDF data extraction, data analysis, and manuscript revision. Umesh Vivekananda contributed to conceptualization, study design, data analysis, and manuscript revision. Nina Moutonnet contributed to EDF anonymization, data curation, and manuscript revision. Sanjeev Rajakulendran and Matthew C. Walker contributed to critical review and revision of the manuscript. All authors approved the final version of the manuscript. Mahinda Yogarajah contributed to the ethical approvals under which this study was conducted.

## CONFLICT OF INTEREST STATEMENT

The authors declare no conflicts of interest. We confirm that we have read the Journal's position on issues involved in ethical publication and affirm that this report is consistent with those guidelines.

## Supporting information


**TABLE S1.** Electroencephalographic variables in the dataset.

**TABLE S2.** Level of consciousness definitions.

## Data Availability

The data that support the findings of this study are available on request from the corresponding author. The data are not publicly available due to privacy or ethical restrictions.
