# Supplementary material for: New onset refractory status epilepticus (NORSE) versus refractory status epilepticus not meeting NORSE criteria: A comparative clinical and electroencephalography‐based study
Source: Epilepsia. 2026 Jul 14;67(10):5437–47. doi: 10.1002/epi.70384 (PMC13675771; doi:10.1002/epi.70384)
Supplement: Supplementary file 1 [file EPI-67-5437-s001.docx]

**aTable2.** LoC definitions.

| **Level of consciousness** | **Description** | **Characteristics** |
| --- | --- | --- |
| **Alert and Oriented** | Patient if fully awake, alert and orientated to person, place and time. | Engages in conversion. Can follow complex instructions and recall recent events. |
| **Following Commands** | Patient can respond to simple verbal stimuli. | Initially may appear drowsy and confused but responds to simple commands. |
| **Other Forms of Responsiveness** | Patient demonstrates some form of non-verbal or non-purposeful response. | Shows reactions such as blinking, grimacing, localising movements in response to stimuli. |
| **Comatose** | Patient is unconscious and does not respond to any external stimuli. | No eye opening, verbal response, or purposeful movement. |
