# Supplementary material for: New onset refractory status epilepticus (NORSE) versus refractory status epilepticus not meeting NORSE criteria: A comparative clinical and electroencephalography‐based study
Source: Epilepsia. 2026 Jul 14;67(10):5437–47. doi: 10.1002/epi.70384 (PMC13675771; doi:10.1002/epi.70384)
Supplement: Supplementary file 2 [file EPI-67-5437-s002.docx]

**aTable1.** EEG variables in the dataset.

| **EEG variables** | **Description** |
| --- | --- |
| ReportTitle | Report title (e.g., 1-06.07.2021-1248) |
| ReportDate | Date of the report (e.g., 2021-07-06) |
| ReportStartTime | Start time of the report (e.g., 1248) |
| Location | Location of the EEG recording (ITU, MITU, Ward, A&E, inpatient, neurophysiology unspecified) |
| Loc | Patient's level of consciousness (alert and orientated, following commands, responsive, unresponsive, unspecified) |
| Interpretation | Interpretation of EEG (abnormal, normal) |
| Slowing | Presence of slow activity (yes, no, unspecified) |
| Encephalopathy | Presence and severity of encephalopathy (yes, mild, moderate, severe, no, unspecified) |
| Seizures | Presence of seizures (yes, no, unspecified) |
| EEG Interpretation: | EEG interpretation (no seizures, liability, seizure, SE, NCSE, no electrical activity) |
| SeizureTimeCourse | Seizure time course (discrete, frequent, continuous) |
| SeizureProportion | Proportion of the recording with continuous activity (whole, partial, unspecified) |
| SeizureCount | Number of seizures within the recording |
| Mean SeizuresDuration | Mean seizure duration in seconds |
| MaximumSeizureLength | Maximum seizure length in seconds |
| Seizure Onset | Seizure onset location (multifocal, left, right, bilateral, unspecified, generalised) |
| SeizureClassification | Clinical seizure observations (subtle motor, motor, tonic-clonic, no, unspecified) |
| ClinicalSeizureObservations | Presence of movement unrelated to seizure (yes, no, unspecified) |
| IEDs | Presence of interictal epileptiform discharges (IEDs) (yes, no, unspecified) |
| IED Type | Type of IEDs (sharp waves, spikes, polyspikes, slow waves, GPDs, PLDs, SIRPIDs) |
| IED Location: | IED location (multifocal, right, unspecified, left, bilateral, non-localising, generalised) |
| ASMs | List of anti-seizure medication (ASMs) used during recording |
| Sedatives | List of sedatives used during recording |
| OtherMedications | List of other medications used (e.g., methylprednisolone, anakinra) |
| Full Clinical Report | Full clinical report for further interpretation and accuracy checking |
| EEGDuration | Duration of the EEG file (minutes) |
| NumberOfSeizuresEEG | Number of seizures recorded in the EEG file |
| SeizuresPerMinute | The number of seizures per minute of EEG duration |
| MeanSeizureDurationEEG | Mean seizure duration in minutes (EEG file) |
| MedianSeizureDurationEEG | Median seizure duration in minutes (EEG file) |
| MaximumSeizureDurationEEG | Maximum seizure duration in minutes (EEG file) |
| EDFFileName | EDF file (e.g., 1-06.07.2021-1248) |
| EDF Date | Date of the EDF recording (e.g., 2021-07-06) |
| EDFStartTime | Start time of the EDF recording (e.g., 1248) |
| NumberOfSeizuresEDF | Number of seizures recorded in the EDF file |
| MeanSeizureDuration | Mean seizure duration in minutes (EDF file) |
| MedianSeizureDuration | Median seizure duration in minutes (EDF file) |
| MaximumSeizureDuration | Maximum seizure duration in minutes (EDF file) |
| EDFDuration | Duration of the EDF file (minutes) |
| Number of Channels | Number of EEG channels used |
| Sampling Rate (Hz): | Sampling rate of the EEG recording (Hz) |
